# Logistic regression with image covariates via the combination of *L*_1_ and Sobolev regularizations

**DOI:** 10.1371/journal.pone.0234975

**Published:** 2020-06-26

**Authors:** Baiguo An, Beibei Zhang

**Affiliations:** School of Statistics, Capital University of Economics and Business, Beijing, China; University of Nevada, Reno, UNITED STATES

## Abstract

The use of image covariates to build a classification model has lots of impact in various fields, such as computer science, medicine, and so on. The aim of this paper is to develop an estimation method for logistic regression model with image covariates. We propose a novel regularized estimation approach, where the regularization is a combination of *L*_1_ regularization and Sobolev norm regularization. The *L*_1_ penalty can perform variable selection, while the Sobolev norm penalty can capture the shape edges information of image data. We develop an efficient algorithm for the optimization problem. We also establish a nonasymptotic error bound on parameter estimation. Simulated studies and a real data application demonstrate that our proposed method performs very well.

## Introduction

As one of the most important issues in machine learning field, classification plays a prominent role throughout various disciplines. Until now people have developed a large number of classification methods, such as KNN, Linear (Quadratic) discriminant analysis, logistic regression, naive bayes, decision tree, SVM, neural network, deep learning, and many others [1, 2]. Among all those methods logistic regression has a long history [3], and is one of the most popular approaches. Logistic regression model is a typical representative of generalized linear models and linear classification methods. Therefore, this article takes logistic regression as the research object. Traditionally, maximum likelihood method is usually used to obtain an estimator of the parameter in logistic regression model [4–6].

However, the big data era brings us massive complex data, one of whose most prominent characteristics is high dimensionality. The maximum likelihood estimation method in logistic regression model faces serious problems such as non-existence, non-uniqueness [7] in high dimensional settings. Regularization is a popular strategy to handle high dimensional problems [8]. Many regularized methods have been proposed over the past decades, including LASSO [9], the smoothly clipped absolute deviation (SCAD) penalty method [10], the minimax concave penalty (MCP) method [11], and so on. For high dimensional logistic regression, [12] considered *L*_1_-regularization path algorithm. [13] proposed an interior-point method for large-scale *L*_1_-regularized logistic regression. [14] proposed the group lasso for logistic regression. The *L*_1/2_ regularized logistic regression is considered by [15] for gene selection in cancer classification.

Image data is a very popular form of data, and generated in many fields, such as computer science, medicine, and so on. In addition to high dimensionality, image data usually contains spatially smooth regions with relatively sharp edges, which leads to its own characteristics including local smoothness [16], jump discontinuity [17], and many others. Local smoothness leads to highly correlated features, which makes the image classification problem more challenging [18]. Jump discontinuity makes conventional smoothing techniques inefficient [17]. On the other hand, using these characteristics in modeling process is often helpful for model efficiency enhancement, and has received a lot of attention recently. For example, [19] introduced a locally adaptive smoothing method for image restoration. [16] proposed Propagation-Separation approach for local likelihood estimation, which can handle local smoothness of image data. [20] developed an adaptive regression model for the analysis of neuroimaging data, which is a generalization of the PS approach. [21] studied theoretical performance of nonlocal means for noise removal of image data. [17] considered a spatially varying coefficient model for neuroimaging data with jump discontinuities. [18] proposed a spatially weighted principal component analysis (SWPCA) for imaging classification. [22] developed a generalized scalar-on-image regression models via total variation regularization, which can keep the piecewise smooth nature of imaging data. [23] proposed an efficient nuclear norm penalized estimation method for matrix linear discriminant analysis.

In this paper, we consider a logistic regression model with image covariates, and develop a regularized estimation approach, which combines the *L*_1_ regularization and the Sobolev norm regularization. The *L*_1_ penalty performs variable selection and removes covariates unrelated to the response from models [9]. The Sobolev norm penalty keeps characteristics of image data (e.g. local smoothness) in model fitting. In fact, the Sobolev regularization is a popular technology in image data analysis, such as image denoising [24], edge detection of images [25], and many others. The proposed regularization method is different from the aforementioned regularized logistic regression models. It is also different from the elastic net method [26], which is a combination of Lasso and ridge regression. The elastic net encourages the grouping effect, where strongly correlated predictors tend to be in or out of the model. However, the elastic net can not exploit structure information of image covariates, and is not suitable for models with image covariates. There are differences between our proposed method and the fused lasso method [27]. In many real data analysis, such as gene expression data, covariates have a order. Adjacent covariates are often highly correlated and have similar effects on the response variable. The fused lasso tends to make adjacent covariates share common effect on the response. The proposed method can be treated as the extended version of the fused lasso from one dimension to multidimensions. Moreover, the fusion term here is Sobolev norm penalty. Furthermore, we develop a novel algorithm to solve the optimization problem. The theoretical property of our estimator is also studied, and a nonasymptotic estimate error bound is given. Numerical studies including simulations and a real data analysis are also considered to verify the performance of our method.

The rest of the article is organized as follows. Section 2 presents the methodology, including model setup, algorithm, and theoretical property. Section 3 is numerical studies, where simulated studies and a real data application are presented. Lastly, we make a short conclusion in Section 4. The proof details of theoretical studies are put in Appendix Section.

## Methodology

### Model setup

Suppose that we have observations (**X**_*i*_, *Y*_*i*_) with 1 ≤ *i* ≤ *n*, where *Y*_*i*_ ∈ {−1, + 1} is the class label, and $X_{i}=\left(x_{jk}^{\left(i\right)}:j=1,\cdots ,p;k=1,\cdots ,q\right)\in R^{p\times q}$ is the corresponding image covariate. We further assume that (**X**_*i*_, *Y*_*i*_) with 1 ≤ *i* ≤ *n* are independent and identically distributed. In order to predict *Y*_*i*_ with **X**_*i*_, the following logistic regression model is assumed

$$\begin{array}{c} \text{log}\frac{P_{i}}{1-P_{i}}=<X_{i},B>, \end{array}$$ (1)

where *P*_*i*_ = *P*(*Y*_*i*_ = +1|**X**_*i*_), $B=\left(b_{jk}\right)\in R^{p\times q}$ is the corresponding coefficient image, and $<X_{i},B>=\sum_{j=1}^{p}\sum_{k=1}^{q}x_{jk}^{\left(i\right)}b_{jk}$ is the inner operator of two matrices. Let *β* = vec(**B**) = (*β*_1_,⋯,*β*_*pq*_)^*T*^ and *X*_*i*_ = vec(**X**_*i*_) = (*x*_*ij*_, *j* = 1,⋯,*pq*)^*T*^ for *i* = 1, ⋯, *n*, then < **X**_*i*_, **B** >= *β*^*T*^ *X*_*i*_, and the model (1) is equivalent to $P_{i}=1/\left(1+e^{-\beta^{T}X_{i}}\right)$. The true value of *β* is denoted by $\beta^{*}={\left(\beta_{1}^{*},\cdots ,\beta_{pq}^{*}\right)}^{T}$.

Traditionally, maximum likelihood method is usually used to estimate coefficient image **B**. The likelihood function is

$$\begin{array}{c} L\left(\beta \right)=\prod_{i=1}^{n}P_{i}^{I(Y_{i}=+1)}{\left(1-P_{i}\right)}^{I(Y_{i}=-1)}=\prod_{i=1}^{n}{\left(1+e^{-Y_{i}\beta^{T}X_{i}}\right)}^{-1}, \end{array}$$

and the corresponding log-likelihood function is $\text{ln}\left(L\left(\beta \right)\right)=-\sum_{i=1}^{n}\text{log}\left(1+e^{-Y_{i}\beta^{T}X_{i}}\right).$ Denote logistic loss function as $l\left(\beta \right)=\text{log}\left(1+e^{-Y_{i}\beta^{T}X_{i}}\right)$, and the associated risk is denoted by Pl(β)=El(β). We assume that $\beta^{*}=\text{arg}\;{\text{min}}_{\beta }Pl\left(\beta \right)$. The empirical risk is denoted by $P_{n}l\left(\beta \right)=n^{-1}\sum_{i=1}^{n}\text{log}\left(1+e^{-Y_{i}\beta^{T}X_{i}}\right)$. Hence, maximizing the likelihood function is equivalent to minimizing the empirical risk

$$\begin{array}{c} \underset{\beta }{\text{min}}\;P_{n}l\left(\beta \right). \end{array}$$

Many optimization methods, such as Newton-Raphson method [6], can be used to solve the above problem.

However, in the image covariate case, the corresponding coefficient image **B** is usually assumed to be a piecewise smooth image with unknown edges. This assumption is widely used in the imaging literature, and is critical for addressing various scientific questions [22]. The maximum likelihood method does not take advantage of these characteristics. Moreover, image covariate is usually high dimensional, and not every element of **X**_*i*_ is useful to predict *Y*_*i*_. But the maximum likelihood method can not perform variable selection. Consequently, we propose a novel estimation method for **B** in the next subsection, which can keep characteristics of image covariate such as local smoothing, and perform variable selection simultaneously.

### Estimation

For the coefficient image **B**, we define its discrete gradient $\nabla B\in R^{p\times q\times 2}$ as

$$\begin{array}{c} {\left(\nabla B\right)}_{jk}=\{\begin{array}{cc} (b_{j+1,k}-b_{j,k},b_{j,k+1}-b_{j,k}), & j<p,k<q,\\ (0,b_{j,k+1}-b_{j,k}), & j=p,k<q,\\ (b_{j+1,k}-b_{j,k},0), & j<p,k=q,\\ (0,0), & j=p,k=q. \end{array} \end{array}$$

(∇**B**)_*jk*_ = (*b*_*j*+1,*k*_ − *b*_*j*,*k*_, *b*_*j*,*k*+1_ − *b*_*j*,*k*_) is the discrete gradient at the position (*j*, *k*). Furthermore, *b*_*j*+1,*k*_ − *b*_*j*,*k*_ is the discrete gradient in the vertical direction, and *b*_*j*,*k*+1_ − *b*_*j*,*k*_ indicates the discrete gradient in the horizontal direction. The Sobolev norm of **B** is the *L*_2_ norm of ∇**B**, which is written as

$$\begin{array}{c} {\left\|B\right\|}_{Sob}=(\sum_{j=1}^{p}\sum_{k=1}^{q}{\left(\nabla B\right)}_{jk}^{2}{)}^{1/2}. \end{array}$$

In fact, we can rewrite ${\left\|B\right\|}_{Sob}^{2}$ as a quadratic form of *β*. Specifically, we define a matrix $D=\left(d_{ij}\right)\in R^{(2pq-p-q)\times pq}$ with *d*_*ij*_ defined in the following formula (2)

$$\begin{array}{c} d_{ij}=\{\begin{array}{cc} -1, & i=(2p-1)(k-1)+s,j=p(k-1)+(s+1)/2,\\ & k=1,\ldots ,(q-1),s=1,3,\ldots ,(2p-3);\\ 1, & i=(2p-1)(k-1)+s,j=p(k-1)+(s+1)/2+1,\\ & k=1,\ldots ,(q-1),s=1,3,\ldots ,(2p-3);\\ -1, & i=(2p-1)(k-1)+s,j=p(k-1)+s/2,\\ & k=1,\ldots ,(q-1),s=2,4,\ldots ,(2p-2);\\ 1, & i=(2p-1)(k-1)+s,j=p(k-1)+s/2+p,\\ & k=1,\ldots ,(q-1),s=2,4,\ldots ,(2p-2);\\ -1, & i=(2p-1)k,j=kp,k=1,\ldots ,(q-1);\\ 1, & i=(2p-1)k,j=kp+p,k=1,\ldots ,(q-1);\\ -1, & i>(2p-1)(q-1),j=i-(p-1)(q-1);\\ 1, & i>(2p-1)(q-1),j=i-(p-1)(q-1)+1,\\ 0, & \text{else}. \end{array} \end{array}$$ (2)

**Fig 1:**
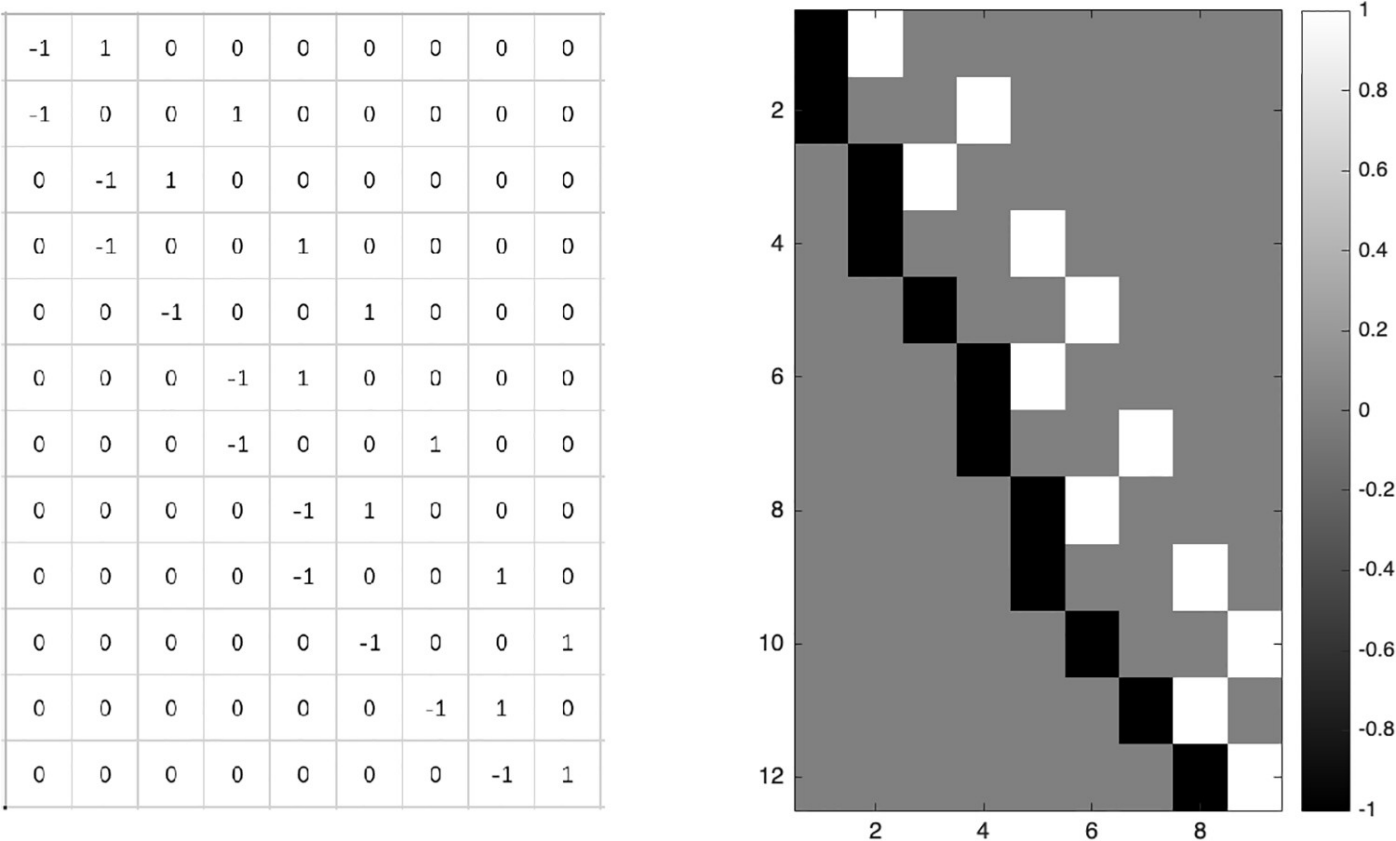
The matrix *D* in the case *p* = *q* = 3.

Then one can easily verify that ${\left\|B\right\|}_{Sob}^{2}=\beta^{T}D^{T}D\beta$. We also present the matrix *D* with a graph in the case *p* = *q* = 3 for the purpose of understanding. Please see Fig (1).

We then consider the following optimization problem

$$\begin{array}{c} \underset{\beta }{\text{min}}\;Q\left(\beta \right), \end{array}$$ (3)

where $Q\left(\beta \right)=P_{n}l\left(\beta \right)+\lambda_{1}\beta^{T}D^{T}D\beta +\lambda_{2}{\left\|\beta \right\|}_{1}$, and ‖⋅‖_1_ is the *L*_1_ norm. The term λ_1_ *β*^*T*^ *D*^*T*^ *Dβ* shrinks adjacent elements of **B** to be similar, hence it can capture the local smoothing of **B**. The term λ_2_‖*β*‖_1_ shrinks the elements of **B** to 0, and performs variable selection. We next propose an algorithm to solve the optimization problem (3).

### Algorithm

For the optimization problem (3), we define *K* = *D*^*T*^ *D* = (*k*_*jl*_) and $H\left(\beta \right)=P_{n}l\left(\beta \right)+\lambda_{1}\beta^{T}K\beta$, then one can see that *Q*(*β*) = *H*(*β*) + λ_2_‖*β*‖_1_. This indicates that the function *Q*(*β*) is a convex function with the separable structure [28]. [29] shows that the coordinate descent algorithm can be guaranteed to converge to the global minimizer for any convex optimization function with the separable structure. Hence we here propose a coordinate descent algorithm to obtain the solution of the optimization problem (3).

For *j* = 1, ⋯, *pq*, we successively minimize *Q*(*β*) along *β*_*j*_ direction with other parameters fixed. Specifically, denote the current value of *β* as *β*^*c*^, and $p_{i}^{c}=P\left(Y_{i}|X_{i},\beta^{c}\right)=1/\left(1+e^{-Y_{i}X_{i}^{T}\beta^{c}}\right)$ for *i* = 1, ⋯, *n*. For *j* = 1, ⋯, *pq*, we use the second order Taylor expansion to approximate function $H(\beta_{-j}^{c},\beta_{j})$ with $\beta_{-j}^{c}={\left(\beta_{1}^{c},\cdots ,\beta_{j-1}^{c},\beta_{j+1}^{c},\cdots ,\beta_{pq}^{c}\right)}^{T}$ fixed. Specifically,

$$\begin{array}{c} \frac{\partial H(\beta_{-j}^{c},\beta_{j})}{\partial \beta_{j}}{|}_{\beta_{j}=\beta_{j}^{c}}=n^{-1}\sum_{i=1}^{n}\left(p_{i}^{c}-1\right)Y_{i}x_{ij}+2\lambda_{1}\sum_{l=1}^{pq}k_{jl}\beta_{l}^{c}, \end{array}$$

$$\begin{array}{c} \frac{\partial^{2}H\left(\beta_{-j}^{c},\beta_{j}\right)}{\partial \beta_{j}^{2}}{|}_{\beta_{j}=\beta_{j}^{c}}=n^{-1}\sum_{i=1}^{n}p_{i}^{c}\left(1-p_{i}^{c}\right)x_{ij}^{2}+2\lambda_{1}k_{jj}. \end{array}$$

Hence,

$$\begin{array}{ccc} H(\beta_{-j}^{c},\beta_{j}) & \approx & H\left(\beta_{-j}^{c},\beta_{j}^{c}\right)+\left(n^{-1}\sum_{i=1}^{n}\left(p_{i}^{c}-1\right)Y_{i}x_{ij}+2\lambda_{1}\sum_{l=1}^{pq}k_{jl}\beta_{l}^{c}\right)\left(\beta_{j}-\beta_{j}^{c}\right)\\ & + & \frac{1}{2}\left(n^{-1}\sum_{i=1}^{n}p_{i}^{c}\left(1-p_{i}^{c}\right)x_{ij}^{2}+2\lambda_{1}k_{jj}\right){\left(\beta_{j}-\beta_{j}^{c}\right)}^{2}. \end{array}$$

Moreover,

$$\begin{array}{ccc} Q(\beta_{-j}^{c},\beta_{j}) & \approx & \left(n^{-1}\sum_{i=1}^{n}\left(p_{i}^{c}-1\right)Y_{i}x_{ij}+2\lambda_{1}\sum_{l=1}^{pq}k_{jl}\beta_{l}^{c}\right)\left(\beta_{j}-\beta_{j}^{c}\right)\\ & + & \frac{1}{2}\left(n^{-1}\sum_{i=1}^{n}p_{i}^{c}\left(1-p_{i}^{c}\right)x_{ij}^{2}+2\lambda_{1}k_{jj}\right){\left(\beta_{j}-\beta_{j}^{c}\right)}^{2}+\lambda_{2}\left|\beta_{j}\right|+C, \end{array}$$

where *C* is a constant containing no information about *β*_*j*_. Denote $\left(n^{-1}\sum_{i=1}^{n}\left(p_{i}^{c}-1\right)Y_{i}x_{ij}+2\lambda_{1}\sum_{l=1}^{pq}k_{jl}\beta_{l}^{c}\right)\left(\beta_{j}-\beta_{j}^{c}\right)+\frac{1}{2}\left(n^{-1}\sum_{i=1}^{n}p_{i}^{c}\left(1-p_{i}^{c}\right)x_{ij}^{2}+2\lambda_{1}k_{jj}\right){\left(\beta_{j}-\beta_{j}^{c}\right)}^{2}+\lambda_{2}\left|\beta_{j}\right|$ by $\tilde{Q}\left(\beta_{j}\right)$. One can update *β*_*j*_ through minimizing $\tilde{Q}\left(\beta_{j}\right)$. Specifically, by

$$\begin{array}{c} \begin{array}{c} \frac{\partial \tilde{Q}\left(\beta_{j}\right)}{\partial \beta_{j}}=\left(n^{-1}\sum_{i=1}^{n}\left(p_{i}^{c}-1\right)Y_{i}x_{ij}+2\lambda_{1}\sum_{l=1}^{pq}k_{jl}\beta_{l}^{c}\right)\\ +\left(n^{-1}\sum_{i=1}^{n}p_{i}^{c}\left(1-p_{i}^{c}\right)x_{ij}^{2}+2\lambda_{1}k_{jj}\right)\left(\beta_{j}-\beta_{j}^{c}\right)+\lambda_{2}\frac{\partial |\beta_{j}|}{\partial \beta_{j}}=0, \end{array} \end{array}$$

where $\frac{\partial |\beta_{j}|}{\partial \beta_{j}}$ is the subderivative, that is $\frac{\partial |\beta_{j}|}{\partial \beta_{j}}=sign\left(\beta_{j}\right)$ if *β*_*j*_ ≠ 0 and $\frac{\partial |\beta_{j}|}{\partial \beta_{j}}\in \left[-1,1\right]$ otherwise, we have that

$$\begin{array}{c} \begin{array}{c} \beta_{j}-\beta_{j}^{c}+{\left(n^{-1}\sum_{i=1}^{n}p_{i}^{c}\left(1-p_{i}^{c}\right)x_{ij}^{2}+2\lambda_{1}k_{jj}\right)}^{-1}\\ \cdot \left(n^{-1}\sum_{i=1}^{n}\left(p_{i}^{c}-1\right)Y_{i}x_{ij}+2\lambda_{1}\sum_{l=1}^{pq}k_{jl}\beta_{l}^{c}\right)=\lambda_{2}{\left(n^{-1}\sum_{i=1}^{n}p_{i}^{c}\left(1-p_{i}^{c}\right)x_{ij}^{2}+2\lambda_{1}k_{jj}\right)}^{-1}\frac{\partial |\beta_{j}|}{\partial \beta_{j}}. \end{array} \end{array}$$

Consequently, one can update *β*_*j*_ as

$$\begin{array}{c} \beta_{j}^{c}\leftarrow \text{sign}\left(\Delta_{j}^{c}\right)(|\Delta_{j}^{c}|-\lambda_{2}{\left(n^{-1}\sum_{i=1}^{n}p_{i}^{c}\left(1-p_{i}^{c}\right)x_{ij}^{2}+2\lambda_{1}k_{jj}\right)}^{-1}{)}_{+}, \end{array}$$

where $\Delta_{j}^{c}=\beta_{j}^{c}-{\left(n^{-1}\sum_{i=1}^{n}p_{i}^{c}\left(1-p_{i}^{c}\right)x_{ij}^{2}+2\lambda_{1}k_{jj}\right)}^{-1}\left(n^{-1}\sum_{i=1}^{n}\left(p_{i}^{c}-1\right)Y_{i}x_{ij}+2\lambda_{1}\sum_{l=1}^{pq}k_{jl}\beta_{l}^{c}\right)$.

We summarize the algorithm as follows.

Coordinate

- Step 1. Initialization. Given initial value *β*.
- Step 2. For *t* = 1, 2, ⋯, update *β*.
  For *j* = 1, ⋯, *p*
  - Compute *p*_*i*_ for 1 ≤ *i* ≤ *n*;
  - Let $\Delta_{j}=\beta_{j}-{\left(n^{-1}\sum_{i=1}^{n}p_{i}\left(1-p_{i}\right)x_{ij}^{2}+2\lambda_{1}k_{jj}\right)}^{-1}\left(n^{-1}\sum_{i=1}^{n}\left(p_{i}-1\right)Y_{i}x_{ij}+2\lambda_{1}\sum_{l=1}^{pq}k_{jl}\beta_{l}\right)$;
  - Update $\beta_{j}\leftarrow \text{sign}\left(\Delta_{j}\right)(|\Delta_{j}|-\lambda_{2}{\left(n^{-1}\sum_{i=1}^{n}p_{i}\left(1-p_{i}\right)x_{ij}^{2}+2\lambda_{1}k_{jj}\right)}^{-1}{)}_{+}$;
  End for.
- Step 3. Repeat Step 2 until convergence.

By the proposed algorithm, we can obtain the solution of (3), which is denoted by $\hat{\beta }$. As the estimator for *β*, the theoretical properties of $\hat{\beta }$ are studied in the next subsection.

### Theoretical properties

In this subsection, we consider the properties of $\hat{\beta }$. A nonasymptotic error bound of $\hat{\beta }$ is given. We assume that the true value β* is sparse. Let $I^{*}=\left\{1\leq j\leq pq:\beta_{j}^{*}\neq 0\right\}$, and $k^{*}=\sum_{j=1}^{pq}I\left(\beta_{j}^{*}\neq 0\right)$ be the cardinality of I*. For the purpose of theoretical studies, we make the following assumptions.

**Assumption 1**. Assume that there exists a constant *L* such that |*x*_*ij*_| ≤ *L* for every 1 ≤ *i* ≤ *n*, 1 ≤ *j* ≤ *pq*.

**Assumption 2**. Assume that there exists a constant *C* such that ‖β*‖_1_ ≤ *C*.

**Assumption 3**. For the matrix *K*, assume that there exists a constant *C*_0_ such that λ_max_(*K*) ≤ *C*_0_, where λ_max_(*K*) is the largest eigenvalue of *K*.

**Assumption 4**. Let $\Sigma =E(X_{i}X_{i}^{T})$. Define the set $V_{\alpha ,\epsilon }=\{\beta \in R^{pq}:\sum_{j\notin I^{*}}|\beta_{j}|\leq \alpha \sum_{j\in I^{*}}\left|\beta_{j}\right|+\epsilon \}\;\text{for}\;\text{some}\;\alpha ,\epsilon .$ Assume that there exists a constant 0 < *b* ≤ 1 such that for every *β* ∈ *V*_*α*, *ϵ*_,

$$\begin{array}{c} P(\beta^{T}\Sigma \beta \geq b\sum_{j\in I^{*}}\beta_{j}^{2}-\epsilon )=1. \end{array}$$

Assumption 1 makes a common bound *L* for all *x*_*ij*_ with *i* = 1, ⋯, *n*, *j* = 1, ⋯, *pq*. Assumption 2 gives a bound for ‖β*‖_1_. Combining Assumptions 1 and 2, one can make sure that *P*_*i*_ with 1 ≤ *i* ≤ *n* are bounded away from zero and one. *P*_*i*_ equalling zero or one will cause the *i*-th subject to be either ignorable or dominant in the likelihood function, that is not expected to appear in statistical analysis. This case can be avoided by Assumptions 1 and 2. In Assumption 3, we assume that the largest eigenvalue of *K* is bounded. Assumption 4 is called Condition Stabil, which can be regarded as a stability requirement on the correlation structure [30]. Under these assumptions, we have the following theorem.

**Theorem 1** *Assume that Assumptions 1-3 are true and Assumption 4 holds for* $\alpha =5,\epsilon =\frac{\text{ln}\;2}{2^{d}}\times \frac{3}{\lambda_{2}}$ *with d* = max{*pq*, *n*}, *let* λ_1_ = λ_2_/(6*CC*_0_), *if*

$$\begin{array}{c} \lambda_{2}\geq 3(7L\sqrt{\frac{2\;\text{ln}\;(2d)}{n}}+\frac{L}{2d}+2L\sqrt{\frac{-2\;\text{ln}\;\delta }{n}}), \end{array}$$ (4)

*then we have that*

$$\begin{array}{c} P(\|\hat{\beta }-\beta^{*}{\|}_{1}\leq \frac{3k^{*}\lambda_{2}}{sb}+\left(1+\frac{3s}{\lambda_{2}}\right)\epsilon )>1-\delta , \end{array}$$

*where* *s* = (1 + *e*^*A*^)^−4^ *with A* = 8*CL is a constant*.

The proof of Theorem 1 is put in the appendix section. The theorem shows that with a high probability, the *L*_1_ norm of estimate error is bounded by 3k*λ_2_/(*sb*) + (1 + 3*s*/λ_2_)*ϵ*. One can see that the term (1 + 3*s*/λ_2_)*ϵ* = *O*(*d*/2^*d*^), which can be negligible for large *d*. Hence, the term 3k*λ_2_/(*sb*) dominates the upper bound, which becomes larger when *b* becomes smaller. If further assume that ln(*pq*) = *o*(*n*), by the condition (4) one can see that λ_2_ can tend to 0. Further 3k*λ_2_/(*sb*) → 0, that means the upper bound can tend to zero. Consequently, the consistency of $\hat{\beta }$ can be guaranteed.

### The selection of tuning parameters

The optimization function (3) contains two tuning parameters λ_1_ and λ_2_, which should be determined by some criteria, such as BIC, cross validation method. In our simulation studies, we select the tuning parameters by a validation set. And in real data analysis, the cross validation method is used. Before applying these methods, one should firstly determine the value range of tuning parameters. Specifically, we here make a transformation of λ_1_ and λ_2_. Let λ = λ_1_ + λ_2_, and *α* = λ_2_/λ. Then the penalty terms in (3) can be rewritten as λ(*α*‖*β*‖_1_ + (1 − *α*)*β*^*T*^ *Kβ*). Because *α* ∈ [0, 1], the alternative values of *α* are set as 0.02*κ* for *κ* = 1, ⋯, 50. With a given *α*, we denote λ_0_ as the threshold value. Once λ ≥ λ_0_, the solution of (3) is exactly zero. By

$$\begin{array}{c} \frac{\partial Q(\beta )}{\partial \beta }=\frac{1}{n}\sum_{i=1}^{n}\frac{e^{-Y_{i}\beta^{T}X_{i}}}{1+e^{-Y_{i}\beta^{T}X_{i}}}\left(-Y_{i}X_{i}\right)+2\lambda \left(1-\alpha \right)K\beta +\lambda \alpha \frac{{\partial \|\beta \|}_{1}}{\beta }=0, \end{array}$$

one can see that once *β* = 0 is the solution, every element of $1/\left(2\lambda \alpha n\right)\sum_{i=1}^{n}\left(-Y_{i}X_{i}\right)$ belongs to [−1, 1]. This means that $\lambda_{0}=1/\left(2\alpha n\right){\left\|\sum_{i=1}^{n}\left(Y_{i}X_{i}\right)\right\|}_{\infty }$. Following the idea of [14], the alternative values of λ are set as 0.001 and 0.96^*ν*^λ_0_ for *ν* = 0, 1, ⋯, 160. For the validation set method, the prediction error on the validation set of our approach with tuning parameters *α*, λ is denoted by *PE*_(*α*, λ)_. The final *α*, λ are selected as the minimizer of *PE*_(*α*, λ)_.

For the *M*-fold cross validation method, the data are randomly divided into *M* folds of approximately equal size. For *m* = 1, ⋯, *M*, we treat the *m* fold as the validation set, and fit the model with tuning parameters *α*, λ on the remaining *M* − 1 flods. The corresponding prediction error on the validation set is denoted by $PE_{\left(\alpha ,\lambda \right)}^{\left(m\right)}$ and the cross validation prediction error is defined as

$$\begin{array}{c} PE_{\left(\alpha ,\lambda \right)}^{\left(cv\right)}=\frac{1}{M}\sum_{m=1}^{M}PE_{\left(\alpha ,\lambda \right)}^{\left(m\right)}. \end{array}$$

The *α*, λ are selected as the minimizer of the cross validation prediction error [6].

## Numerical studies

In this section, we evaluate the performance of our proposed method by two simulated examples and a real data analysis. For the purpose of comparison, we also consider the logistic regression model with *L*_1_ penalty [12, 13], the logistic regression model with fused lasso penalty, and linear support vector machine, which are denoted by LG-*L*_1_, LG-fused, and Linear SVM respectively for convenience. Meanwhile, our proposed method is abbreviated as LG-sob.

### Simulation studies

**Fig 2:**
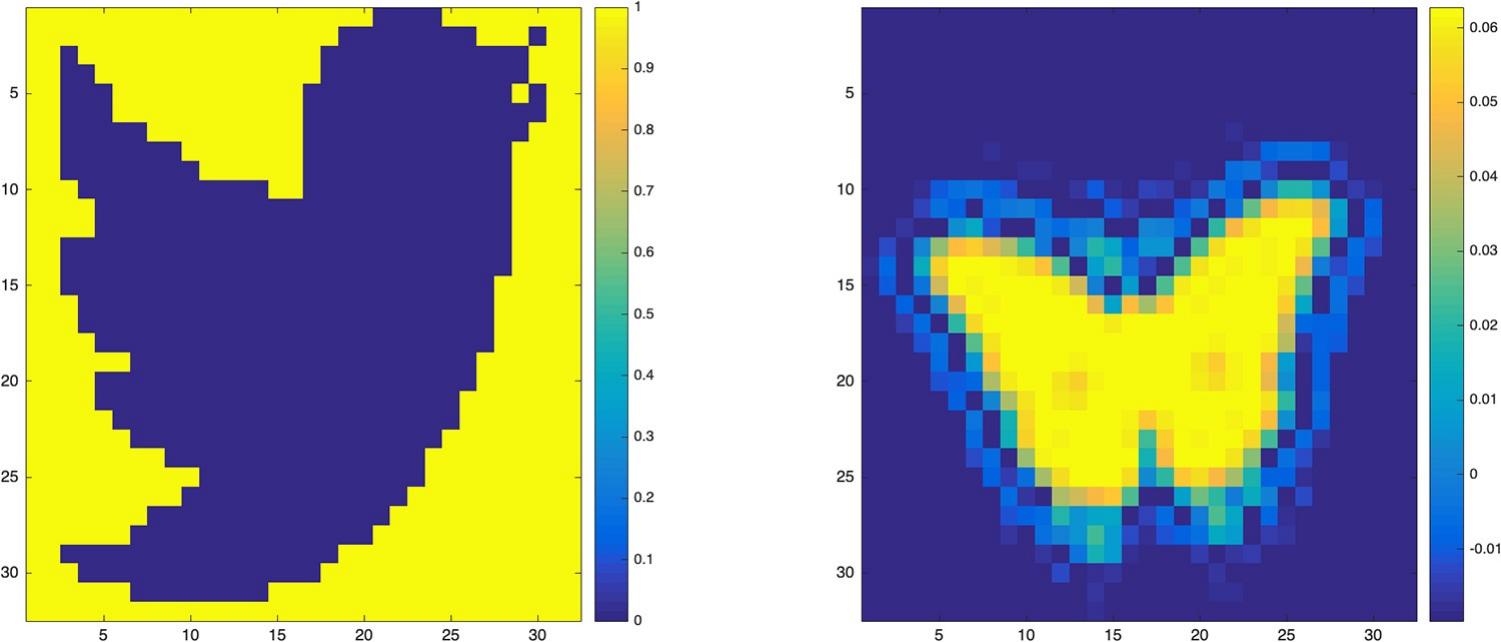
Simulated example. The true parameter images *B*_0_.

**Example 1**. We generate data from the following model

$$\begin{array}{c} \text{log}\frac{P(Y_{i}=+1|X_{i})}{P(Y_{i}=-1|X_{i})}=<X_{i},B_{0}>,\;\;i=1,\cdots ,n, \end{array}$$

where **X**_*i*_ and *B*_0_ both belong to $R^{32\times 32}$. One result caused by image covariates is that the corresponding regression coefficient can be treated as a image too. Hence we here just treat *B*_0_ as images, while **X**_*i*_ is generated from a multivariate normal distribution. Specifically, we define the vectorization of **X**_*i*_ as *X*_*i*_, and *X*_*i*_ is generated from a multivariate normal distribution with mean 0 and covariance $\text{cov}\left(x_{ij_{1}},x_{ij_{2}}\right)=0.5^{|j_{1}-j_{2}|}$ for any 1 ≤ *j*_1_, *j*_2_ ≤ 1024. The parameter image *B*_0_ is considered in two cases, which have been shown in Fig 2. The first case of *B*_0_ denoted by *B*_01_ is a bird picture, in which the blue region takes value 0, and the yellow region takes value 1. The other case of *B*_0_ denoted by *B*_02_ is a butterfly picture, which is more complicated and takes values in interval [−0.0197, 0.0628]. Given **X**_*i*_ and *B*_0_, the response *Y*_*i*_ is generated from a two-point distribution $P\left(Y_{i}=+1|X_{i}\right)=1-P\left(Y_{i}=-1|X_{i}\right)=1/\left(1+e^{-<X_{i},B_{0}>}\right)$.

**Example 2**. In this example, the mechanism of data generation is similar to that for Example 1, the only difference is that we generate **X**_*i*_ in a more complex way. In particular, we follow the simulation scheme of [22] and generate **X**_*i*_ from a 32 × 32 phantom map with 1024 pixels according to a spatially correlated random process *X*_*i*_ = ∑_*l*_ *l*^−1^ *η*_*il*_ *φ*_*l*_, among which the *η*_*il*_ are standard normal random variables and the *φ*_*l*_ are bivariate Haar wavelet basis functions.

For these two simulated examples, along with the training set with sample size *n*, we also generate a validation set and a test set with sample sizes both being 500. We train the model on the training set, select tuning parameters through the validation set, and calculate the classification accuracy on the test set to evaluate the performance of the model.

For every specification of the parameter *B*_0_ and sample size *n*, we replicate the simulation 100 times for each example, and the average prediction errors are computed and summarized in Table 1 for Example 1, and Table 2 for Example 2 respectively. Besides the prediction errors, we also calculate the average estimation errors $\sum_{i=1}^{100}{\left\|{\hat{B}}_{i}-B_{0}\right\|}^{2}/100$ for LG-sob, LG-*L*_1_ and LG-fused, where ${\hat{B}}_{i}$ is the parameter image estimator in the *i*-th time. From the results, one can see that our proposed method performs better than the other three methods in all cases from the prediction perspective. As sample size *n* becomes larger, the prediction errors will become smaller, but the estimation errors do not decrease congruously. The reason may be that the tuning parameters are selected based on minimization of prediction error.

**Table 1:** Results of simulated example 1: Prediction error (PE) and estimation error (EE).

(*n*, *B*_0_)	(500, *B*_01_)		(1000, *B*_01_)		(500, *B*_02_)		(1000, *B*_02_)	
	PE	EE	PE	EE	PE	EE	PE	EE
LG-sob	0.099	337.645	0.075	336.173	0.107	8.153	0.080	13.505
LG-*L*_1_	0.272	404.589	0.199	375.926	0.272	17.354	0.204	23.143
LG-fused	0.248	423.242	0.190	406.866	0.248	7.016	0.190	9.400
Linear SVM	0.221	NA	0.174	NA	0.223	NA	0.172	NA

**Table 2:** Results of simulated example 2: Prediction error (PE) and estimation error (EE).

(*n*, *B*_0_)	(500, *B*_01_)		(1000, *B*_01_)		(500, *B*_02_)		(1000, *B*_02_)	
	PE	EE	PE	EE	PE	EE	PE	EE
LG-sob	0.028	181.06	0.023	176.19	0.049	582.94	0.038	1096.1
LG-*L*_1_	0.073	1712.6	0.058	1501.7	0.116	2190.9	0.090	3022.4
LG-fused	0.052	438.57	0.044	495.73	0.097	482.19	0.076	775.99
Linear SVM	0.050	NA	0.038	NA	0.084	NA	0.071	NA

**Fig 3:**
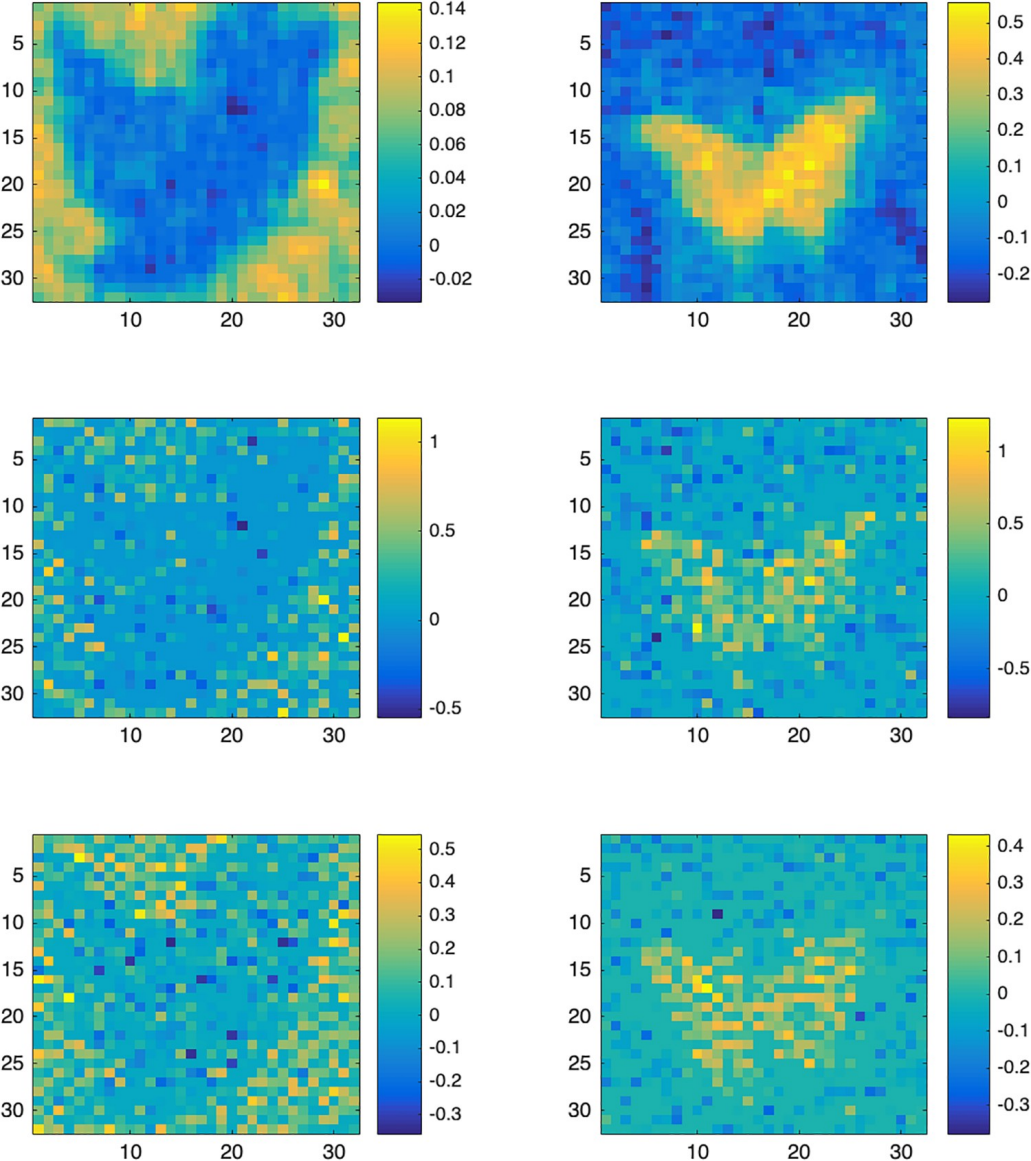
Simulated example. One of randomly selected parameter images estimations. The first row is the results of our proposed LG-sob, the second row is the results of LG-*L*_1_, the third row is the results of LG-fused.

Moreover, we also randomly select one simulated result from the 100 replications of Example 1, and show the parameter image estimations in Fig 3. One can see that our proposed LG-sob method can capture the shapes of images, but LG-*L*_1_ and LG-fused do not have this property.

### A real data analysis

**Fig 4:**
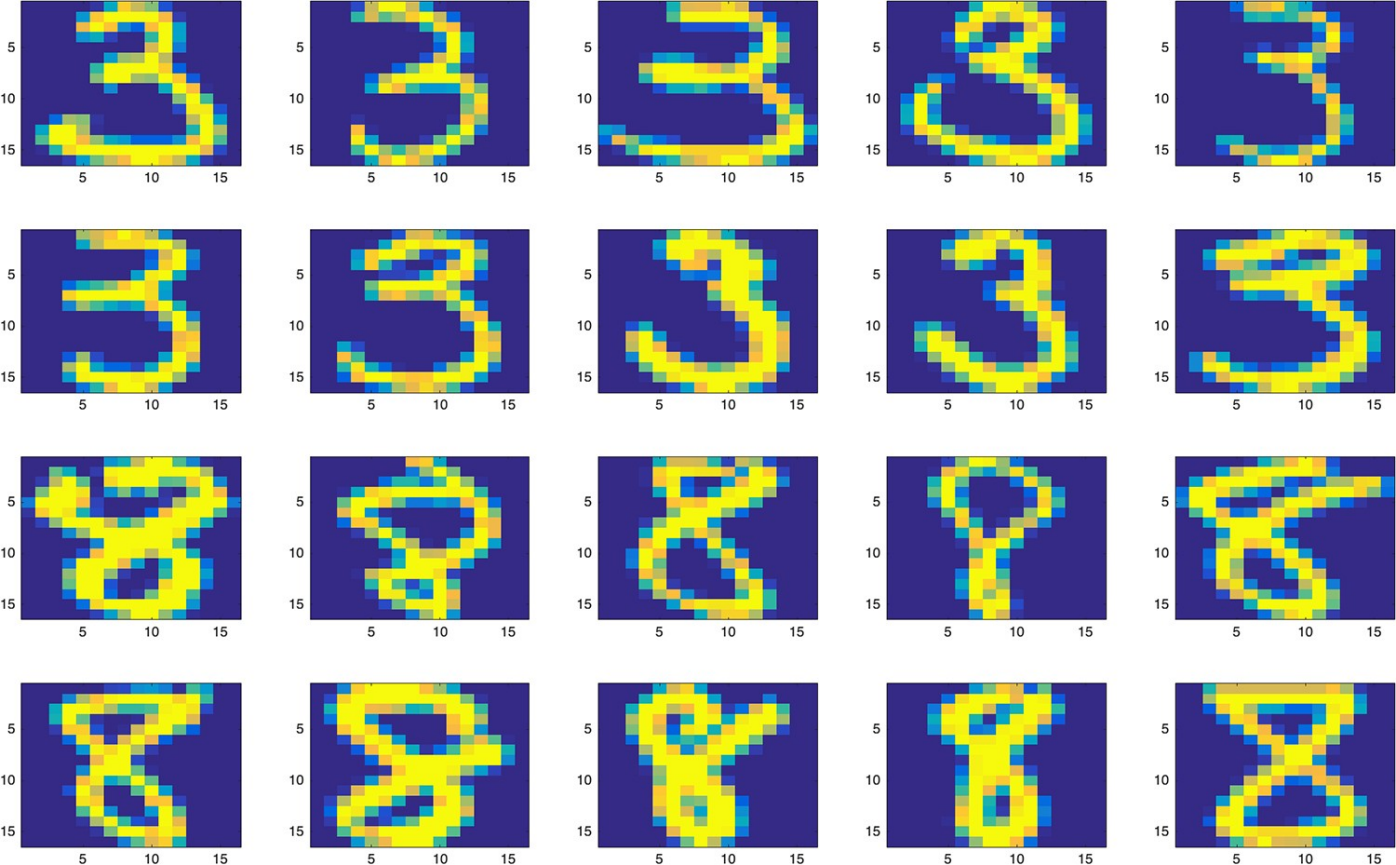
Real data analysis. Some examples of handwritten 3’s and 8’s.

The classification of the ZIP Code Dataset is a benchmark problem in machine learning community [6]. One can obtain the ZIP Code Dataset from the following website https://web.stanford.edu/~hastie/StatLearnSparsity_files/DATA/zipcode.html [28]. The Dataset contains normalized handwritten digits, which are automatically scanned from envelopes by the U.S. Postal Service. Every observation is a handwritten digit, and is represented as a size normalized 16 × 16 grayscale image [31]. The purpose is to use the 256 pixel values to predict the corresponding digit. The Dataset contains a training set with 7291 observations and a test set with 2007 observations. Because this article only considers the binary response prediction by logistic regression models, and it looks like that numbers 3 and 8 have more similar characteristics, hence we only consider handwritten 3’s and 8’s in this paper. The sizes of handwritten 3’s and 8’s are 658 and 542 respectively in the train set, while they are both 166 in the test set. Fig 4 shows some examples of handwritten 3’s and 8’s.

More specifically, we denote the *i*-th observation by $X_{i}\in R^{16\times 16}$, and define the corresponding class label *Y*_*i*_ = −1 if **X**_*i*_ represents handwritten 3 and *Y*_*i*_ = + 1 if **X**_*i*_ represents handwritten 8. Our proposed method is applied to construct the classifier for the prediction of *Y*_*i*_ (i.e. handwritten numeral) based on the grayscale image **X**_*i*_. We train the model on the training set, and evaluate the performance of the proposed method on the test set by classification accuracy. For the purpose of comparison, we also consider the logistic regression model with only *L*_1_ penalty.

**Fig 5:**
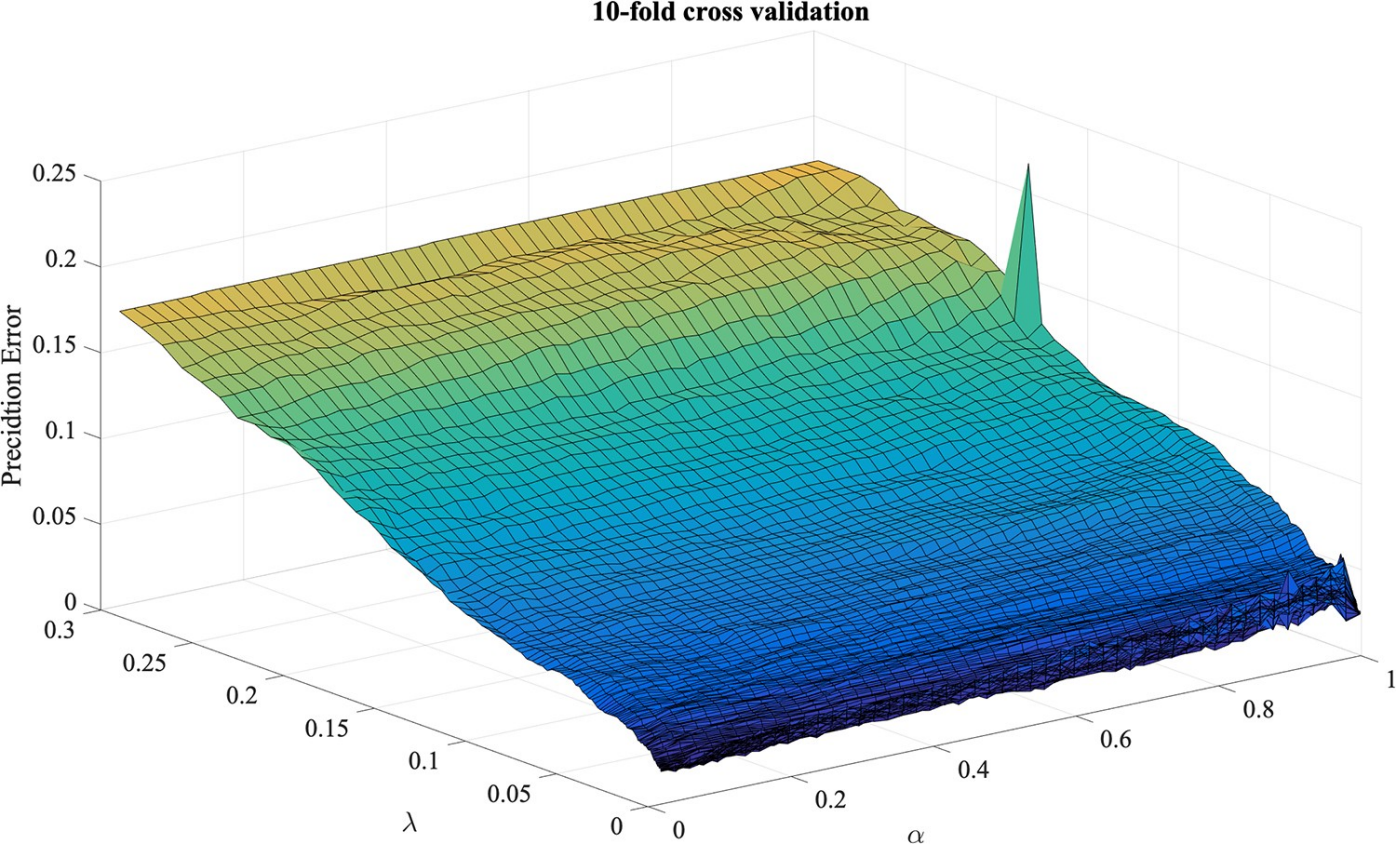
Real data analysis. The results of 10-fold CV: Prediction error in various parameters settings.

**Fig 6:**
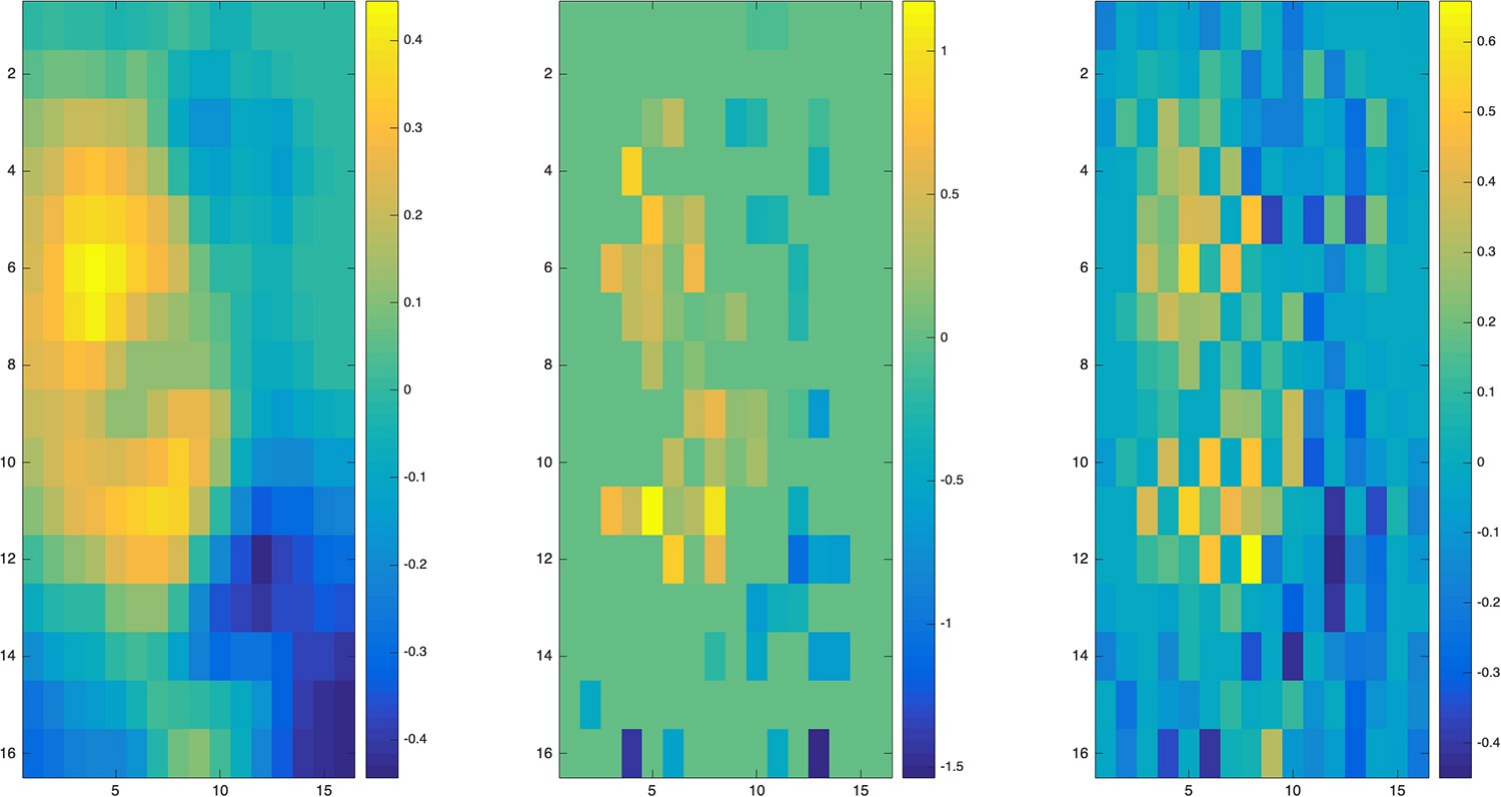
Real data analysis. The parameter image estimations. Left: the estimation of our proposed LG-sob; Middle: the estimation of LG-*L*_1_; Right: the estimation of LG-fused.

The tuning parameters are selected by 10-fold cross validation (CV) method. The CV prediction errors in various parameters setting are calculated and plotted in Fig 5. Finally, our proposed method selects the tuning parameters as *α* = 0.04, λ = 0.0118, while the method with *L*_1_ penalty selects the tuning parameter as λ = 0.0014. The parameter image estimations of the two methods are shown in Fig 6. One can see that our proposed method tends to make adjacent pixels have similar effects on the model. Meanwhile, LG-*L*_1_ tries to obtain a more sparse parameter estimation, and LG-fused method tries to make pixels only adjacent in the vertical direction have similar effects. The top-left region of parameter image has positive effects on handwritten numeral 8, and the bottom-right region has positive effects on handwritten numeral 3. The classification accuracy on the test set of our proposed method is 96.99%, while the accuracies of LG-*L*_1_, LG-fused, and Linear SVM are 96.39%, 96.08% and 96.39%, respectively. The proposed method performs better.

## Conclusion

We have developed a novel estimation method for logistic regression with image covariates. This method can not only perform variable selection, but also capture the shape features of the parameter images. Both theoretical results and numerical studies show that our method performs well. We have proposed a coordinated descent algorithm to solve the optimization problem, and the global convergence of the algorithm is guaranteed. However, as pointed out by one referee, the coordinated descent algorithm is very time consuming, especially in the case of high dimensional image covariates. Many more efficient optimalization approaches, such as Nesterov accelerated gradient methods [32], interior-point methods [13], may be more suitable. We will research this issue in future. Furthermore, our method is mainly based on Sobolev norm regularization, compared to which total variation regularization is more sensitive to capture sharp edges and jumps of parameter images. However, the algorithm of total variation regularization based estimation method is more complex, which can be our future work to study.

## Appendix: Proof of Theorem 1

Before giving the proof of Theorem 1, we first list the bounded differences inequality as the following lemma without proof.

**Lemma 1** *(the Bounded Differences Inequality) Suppose that* $X_{1},\cdots ,X_{n}\in H$ *are independent, and the function* $f:H^{n}\to R$ *satisfies the bounded difference assumption*

$$\begin{array}{c} \underset{x_{1},\cdots ,x_{n},x_{i}^{{}^{\prime }}\in H}{\sup}\left|f\left(x_{1},\cdots ,x_{n}\right)-f\left(x_{1},\cdots ,x_{i-1},x_{i}^{{}^{\prime }},x_{i+1},\cdots ,x_{n}\right)\right|\leq c_{i}, \end{array}$$

for *i* = 1, ⋯, *n*. Then for all *t* > 0,

$$\begin{array}{c} P\left(f-E\left(f\right)\geq t\right)\leq e^{-2t^{2}/\sum_{i=1}^{n}c_{i}^{2}}. \end{array}$$

For more details about Lemma 1 and its proof, one can refer to [33]. The following is the proof of Theorem 1.

**Proof of Theorem 1**. By the definitions of $\hat{\beta }$ and β*, one can see that

$$\begin{array}{c} Pl\left(\hat{\beta }\right)\geq Pl\left(\beta^{*}\right), \end{array}$$

and

$$\begin{array}{c} P_{n}l\left(\hat{\beta }\right)+\lambda_{1}{\hat{\beta }}^{T}K\hat{\beta }+\lambda_{2}\|\hat{\beta }{\|}_{1}\leq P_{n}l\left(\beta^{*}\right)+\lambda_{1}\beta^{*T}K\beta^{*}+\lambda_{2}{\left\|\beta^{*}\right\|}_{1}. \end{array}$$

Hence, we have that

$$\begin{array}{ccc} 0 & \leq & Pl\left(\hat{\beta }\right)-Pl\left(\beta^{*}\right)\\ & \leq & \left(P_{n}-P\right)\left(l\left(\beta^{*}\right)-l\left(\hat{\beta }\right)\right)+\lambda_{1}\left(\beta^{*T}K\beta^{*}-{\hat{\beta }}^{T}K\hat{\beta }\right)+\lambda_{2}\left(\right\|\beta^{*}{\|}_{1}-\|\hat{\beta }{\|}_{1}). \end{array}$$ (5)

Moreover,

$$\begin{array}{ccc} & & \lambda_{2}/3{\left\|\hat{\beta }-\beta^{*}\right\|}_{1}\\ & \leq & \lambda_{2}/3{\left\|\hat{\beta }-\beta^{*}\right\|}_{1}+\lambda_{1}{\left(\hat{\beta }-\beta^{*}\right)}^{T}K\left(\hat{\beta }-\beta^{*}\right)\\ & \leq & \lambda_{2}/3{\left\|\hat{\beta }-\beta^{*}\right\|}_{1}+\lambda_{1}{\left(\hat{\beta }-\beta^{*}\right)}^{T}K\left(\hat{\beta }-\beta^{*}\right)+Pl\left(\hat{\beta }\right)-Pl\left(\beta^{*}\right)\\ & \leq & \lambda_{2}/3{\left\|\hat{\beta }-\beta^{*}\right\|}_{1}+\lambda_{1}{\left(\hat{\beta }-\beta^{*}\right)}^{T}K\left(\hat{\beta }-\beta^{*}\right)+\lambda_{1}\left(\beta^{*T}K\beta^{*}-{\hat{\beta }}^{T}K\hat{\beta }\right)\\ & & +\left(P_{n}-P\right)\left(l\left(\beta^{*}\right)-l\left(\hat{\beta }\right)\right)+\lambda_{2}\left(\right\|\beta^{*}{\|}_{1}-\|\hat{\beta }{\|}_{1}). \end{array}$$ (6)

We first consider the term $\left(P_{n}-P\right)\left(l\left(\beta^{*}\right)-l\left(\hat{\beta }\right)\right)$. Specifically, define $L_{n}=\sup_{\beta }\frac{\left(P_{n}-P\right)\left(l\left(\beta^{*}\right)-l\left(\beta \right)\right)}{\|\beta -\beta^{*}{\|}_{1}+\epsilon }$. Let $l\left(\beta ;Y_{i},X_{i}\right)=\text{log}\left(1+e^{-Y_{i}\beta^{T}X_{i}}\right)$, and

$$\begin{array}{c} P_{nl}l\left(\beta \right)=\frac{1}{n}\left(\sum_{i=1,i\neq l}^{n}l\left(\beta ;Y_{i},X_{i}\right)+l\left(\beta ;Y_{l}^{{}^{\prime }},X_{l}^{{}^{\prime }}\right)\right), \end{array}$$

which is the empirical measure corresponding to replacing (*Y*_*l*_, *X*_*l*_) by $\left(Y_{l}^{{}^{\prime }},X_{l}^{{}^{\prime }}\right)$. Then

$$\begin{array}{ccc} & & \frac{\left(P_{n}-P\right)\left(l\left(\beta^{*}\right)-l\left(\beta \right)\right)}{\|\beta -\beta^{*}{\|}_{1}+\epsilon }-\frac{\left(P_{nl}-P\right)\left(l\left(\beta^{*}\right)-l\left(\beta \right)\right)}{\|\beta -\beta^{*}{\|}_{1}+\epsilon }\\ & = & \frac{1}{n}\frac{l\left(\beta^{*};Y_{l},X_{l}\right)-l\left(\beta ;Y_{l},X_{l}\right)-l\left(\beta^{*};Y_{l}^{{}^{\prime }},X_{l}^{{}^{\prime }}\right)+l\left(\beta ;Y_{l}^{{}^{\prime }},X_{l}^{{}^{\prime }}\right)}{\|\beta -\beta^{*}{\|}_{1}+\epsilon }\\ & \leq & \frac{4L}{n}\frac{\|\beta -\beta^{*}{\|}_{1}}{\|\beta -\beta^{*}{\|}_{1}+\epsilon }\leq \frac{4L}{n}, \end{array}$$

among which the inequality is obtained by a first order Taylor expansion and the assumption 1. Then by Lemma 1, we can obtain that

$$\begin{array}{c} P\left(L_{n}-E\left(L_{n}\right)\geq u\right)\leq \text{exp}\left\{-\frac{nu^{2}}{8L^{2}}\right\}. \end{array}$$

Let $\delta =\text{exp}\{-\frac{nu^{2}}{8L^{2}}\}$, then we have that $u=2L\sqrt{\frac{-2\text{ln}\;\delta }{n}}$, and *P*(*L*_*n*_ − *E*(*L*_*n*_) ≥ *u*) ≤ *δ*.

Let *d* = max{*pq*, *n*}. Taking $\epsilon =\frac{\text{ln}\;2}{2^{d}}\times \frac{3}{\lambda_{2}}$, by the lemma 3 of [34] with *C*_*φ*_ = 1, *C*_*F*_ = *L*, we have

$$\begin{array}{c} E\left(L_{n}\right)\leq 7L\sqrt{\frac{2\;\text{ln}(2d)}{n}}+\frac{L}{2d}. \end{array}$$

Consequently, we have that

$$\begin{array}{c} P(L_{n}\leq 7L\sqrt{\frac{2\;\text{ln}\;(2d)}{n}}+\frac{L}{2d}+2L\sqrt{\frac{-2\;\text{ln}\;\delta }{n}})\geq 1-\delta . \end{array}$$

By the condition of Theorem 1, we know $\lambda_{2}\geq 3(7L\sqrt{\frac{2\;\text{ln}\;(2d)}{n}}+\frac{L}{2d}+2L\sqrt{\frac{-2\;\text{ln}\;\delta }{n}})$, hence *P*(*L*_*n*_ ≤ λ_2_/3) ≥ 1 − *δ*.

On the event {*L*_*n*_ ≤ λ_2_/3}, we have that

$$\begin{array}{c} \left(P_{n}-P\right)\left(l\left(\beta^{*}\right)-l\left(\hat{\beta }\right)\right)\leq \frac{\lambda_{2}}{3}(\|\beta -\beta^{*}{\|}_{1}+\epsilon ). \end{array}$$ (7)

Secondly, we consider the term $\lambda_{1}{\left(\hat{\beta }-\beta^{*}\right)}^{T}K\left(\hat{\beta }-\beta^{*}\right)+\lambda_{1}\left(\beta^{*T}K\beta^{*}-{\hat{\beta }}^{T}K\hat{\beta }\right)$. Based on Assumptions 2 and 3, one can see that

$$\begin{array}{ccc} & & \lambda_{1}{\left(\hat{\beta }-\beta^{*}\right)}^{T}K\left(\hat{\beta }-\beta^{*}\right)+\lambda_{1}\left(\beta^{*T}K\beta^{*}-{\hat{\beta }}^{T}K\hat{\beta }\right)\\ & = & 2\lambda_{1}\beta^{*T}K\left(\beta^{*}-\hat{\beta }\right)\\ & \leq & 2\lambda_{1}\lambda_{\max}\left(K\right)\|\beta^{*}{\|}_{2}{\left\|\hat{\beta }-\beta^{*}\right\|}_{2}\\ & \leq & 2\lambda_{1}\lambda_{\max}\left(K\right)\|\beta^{*}{\|}_{1}{\left\|\hat{\beta }-\beta^{*}\right\|}_{1}\\ & \leq & 2\lambda_{1}CC_{0}{\left\|\hat{\beta }-\beta^{*}\right\|}_{1}. \end{array}$$

One can see that if λ_1_ = λ_2_/(6*CC*_0_), we have

$$\begin{array}{c} \lambda_{1}{\left(\hat{\beta }-\beta^{*}\right)}^{T}K\left(\hat{\beta }-\beta^{*}\right)+\lambda_{1}\left(\beta^{*T}K\beta^{*}-{\hat{\beta }}^{T}K\hat{\beta }\right)\leq \lambda_{2}/3{\left\|\hat{\beta }-\beta^{*}\right\|}_{1}. \end{array}$$ (8)

Consequently, on the event {*L*_*n*_ ≤ λ_2_/3} we combine (6), (7), (8), and obtain

$$\begin{array}{ccc} \lambda_{2}/3{\left\|\hat{\beta }-\beta^{*}\right\|}_{1} & \leq & \lambda_{2}\|\hat{\beta }-\beta^{*}{\|}_{1}+\lambda_{2}\left(\right\|\beta^{*}{\|}_{1}-\|\hat{\beta }{\|}_{1})+\lambda_{2}/3\epsilon \\ & \leq & \lambda_{2}\left(\right\|\hat{\beta }{\|}_{1}+\|\beta^{*}{\|}_{1})+\lambda_{2}\left(\right\|\beta^{*}{\|}_{1}-\|\hat{\beta }{\|}_{1})+\lambda_{2}/3\epsilon \\ & \leq & 2\lambda_{2}{\left\|\beta^{*}\right\|}_{1}+\lambda_{2}/3\epsilon . \end{array}$$ (9)

Hence we have that

$$\begin{array}{c} \|\hat{\beta }-\beta^{*}{\|}_{1}\leq 6{\left\|\beta^{*}\right\|}_{1}+\epsilon \leq 7C. \end{array}$$ (10)

By (9) one can also obtain that

$$\begin{array}{ccc} \|\hat{\beta }-\beta^{*}{\|}_{1} & \leq & 3\|\hat{\beta }-\beta^{*}{\|}_{1}+3(\|\beta^{*}{\|}_{1}-\|\hat{\beta }{\|}_{1})+\epsilon \\ & = & 3(\sum_{j\in I^{*}}|{\hat{\beta }}_{j}-\beta_{j}^{*}|+\sum_{j\notin I^{*}}|{\hat{\beta }}_{j}|+\sum_{j\in I^{*}}|\beta_{j}^{*}|-\sum_{j=1}^{pq}|{\hat{\beta }}_{j}|)+\epsilon \\ & = & 3(\sum_{j\in I^{*}}|{\hat{\beta }}_{j}-\beta_{j}^{*}|+\sum_{j\in I^{*}}|\beta_{j}^{*}|-\sum_{j\in I^{*}}|{\hat{\beta }}_{j}|)+\epsilon \\ & \leq & 6\sum_{j\in I^{*}}\left|{\hat{\beta }}_{j}-\beta_{j}^{*}\right|+\epsilon . \end{array}$$

Consequently, we have $\sum_{j\notin I^{*}}|{\hat{\beta }}_{j}-\beta_{j}^{*}|\leq 5\sum_{j\in I^{*}}\left|{\hat{\beta }}_{j}-\beta_{j}^{*}\right|+\epsilon$. This means that $\hat{\beta }-\beta^{*}\in V_{5,\epsilon }$.

By the example 4.5 in [35], we have that $Pl\left(\hat{\beta }\right)-Pl\left(\beta^{*}\right)\geq E_{X}{\left(P\left(\hat{\beta }\right)-P\left(\beta^{*}\right)\right)}^{2}$, where *P*(*β*) = 1/(1+ *e*^−*X*^*T*^*β*^) and *E*_*X*_(⋅) is the expectation with respect to the distribution of *X*. Using Taylor expansion, one can obtain that

$$\begin{array}{c} P\left(\hat{\beta }\right)-P\left(\beta^{*}\right)=\frac{e^{{\tilde{\beta }}^{T}X}}{{\left(1+e^{{\tilde{\beta }}^{T}X}\right)}^{2}}X^{T}\left(\hat{\beta }-\beta^{*}\right), \end{array}$$

where $\tilde{\beta }=\tau \hat{\beta }+\left(1-\tau \right)\beta^{*}$ for some *τ* ∈ (0, 1). Moreover, by (10) and Assumptions 1-2, we have ${\tilde{\beta }}^{T}X\leq \|\tilde{\beta }{\|}_{1}L\leq \left(\tau \right\|\hat{\beta }-\beta^{*}{\|}_{1}+\|\beta^{*}{\|}_{1})L\leq 8CL.$ This means that

$$\begin{array}{c} {\left(P\left(\hat{\beta }\right)-P\left(\beta^{*}\right)\right)}^{2}\geq s{\left(\hat{\beta }-\beta^{*}\right)}^{T}XX^{T}\left(\hat{\beta }-\beta^{*}\right), \end{array}$$

where *s* = (1 + *e*^*A*^)^−4^ and *A* = 8*CL*, then by Assumption 4 we have that

$$\begin{array}{c} Pl\left(\hat{\beta }\right)-Pl\left(\beta^{*}\right)\geq E_{X}{\left(P\left(\hat{\beta }\right)-P\left(\beta^{*}\right)\right)}^{2}\geq s{\left(\hat{\beta }-\beta^{*}\right)}^{T}\Sigma \left(\hat{\beta }-\beta^{*}\right)\geq sb\sum_{j\in I^{*}}{\left(\hat{\beta_{j}}-\beta_{j}^{*}\right)}^{2}-s\epsilon . \end{array}$$ (11)

Furthermore, we have

$$\begin{array}{ccc} & & \frac{\lambda_{2}}{3}{\left\|\hat{\beta }-\beta^{*}\right\|}_{1}+sb\sum_{j\in I^{*}}{\left(\hat{\beta_{j}}-\beta_{j}^{*}\right)}^{2}-s\epsilon \\ & \leq & \frac{\lambda_{2}}{3}{\left\|\hat{\beta }-\beta^{*}\right\|}_{1}+Pl\left(\hat{\beta }\right)-Pl\left(\beta^{*}\right)\\ & \leq & \lambda_{2}/3{\left\|\hat{\beta }-\beta^{*}\right\|}_{1}+\lambda_{1}\left(\beta^{*T}K\beta^{*}-{\hat{\beta }}^{T}K\hat{\beta }\right)+\left(P_{n}-P\right)\left(l\left(\beta^{*}\right)-l\left(\hat{\beta }\right)\right)\\ & & +\lambda_{2}\left(\right\|\beta^{*}{\|}_{1}-\|\hat{\beta }{\|}_{1})\\ & \leq & \lambda_{2}/3{\left\|\hat{\beta }-\beta^{*}\right\|}_{1}+\lambda_{1}{\left(\hat{\beta }-\beta^{*}\right)}^{T}K\left(\hat{\beta }-\beta^{*}\right)+\lambda_{1}\left(\beta^{*T}K\beta^{*}-{\hat{\beta }}^{T}K\hat{\beta }\right)\\ & & +\left(P_{n}-P\right)\left(l\left(\beta^{*}\right)-l\left(\hat{\beta }\right)\right)+\lambda_{2}\left(\right\|\beta^{*}{\|}_{1}-\|\hat{\beta }{\|}_{1})\\ & \leq & \lambda_{2}\|\hat{\beta }-\beta^{*}{\|}_{1}+\lambda_{2}\left(\right\|\beta^{*}{\|}_{1}-\|\hat{\beta }{\|}_{1})+\lambda_{2}/3\epsilon \\ & = & \lambda_{2}(\sum_{j\in I^{*}}|{\hat{\beta }}_{j}-\beta_{j}^{*}|+\sum_{j\notin I^{*}}|\hat{\beta_{j}}\left|\right)+\lambda_{2}(\sum_{j\in I^{*}}|\beta_{j}^{*}|-\sum_{j\in I^{*}}|\hat{\beta_{j}}|-\sum_{j\notin I^{*}}|\hat{\beta_{j}}\left|\right)\\ & & +\lambda_{2}/3\epsilon \\ & = & \lambda_{2}\sum_{j\in I^{*}}|{\hat{\beta }}_{j}-\beta_{j}^{*}|+\lambda_{2}(\sum_{j\in I^{*}}|\beta_{j}^{*}|-\sum_{j\in I^{*}}|\hat{\beta_{j}}\left|\right)+\lambda_{2}/3\epsilon \\ & \leq & 2\lambda_{2}\sum_{j\in I^{*}}\left|{\hat{\beta }}_{j}-\beta_{j}^{*}\right|+\lambda_{2}/3\epsilon , \end{array}$$

where the first inequality follows by (11), the second inequality follows by (5), and the fourth inequality is obtained by combining the results of (7) and (8). Consequently, we have

$$\begin{array}{c} \|\hat{\beta }-\beta^{*}{\|}_{1}+\frac{3}{\lambda_{2}}sb\sum_{j\in I^{*}}{\left(\hat{\beta_{j}}-\beta_{j}^{*}\right)}^{2}\leq 6\sum_{j\in I^{*}}\left|\hat{\beta_{j}}-\beta_{j}^{*}\right|+\left(1+\frac{3s}{\lambda_{2}}\right)\epsilon \\ \leq 9ak^{*}+\frac{1}{a}\sum_{j\in I^{*}}{\left(\hat{\beta_{j}}-\beta_{j}^{*}\right)}^{2}+\left(1+\frac{3s}{\lambda_{2}}\right)\epsilon , \end{array}$$

where *a* is a positive constant and the second inequality follows by

$$\begin{array}{c} 6|\hat{\beta_{j}}-\beta_{j}^{*}|=2\cdot 3a^{1/2}\cdot a^{-1/2}\left|\hat{\beta_{j}}-\beta_{j}^{*}\right|\leq 6a+{\left(\hat{\beta_{j}}-\beta_{j}^{*}\right)}^{2}/a. \end{array}$$

Let *a* = λ_2_/(3*sb*), then

$$\begin{array}{c} \|\hat{\beta }-\beta^{*}{\|}_{1}\leq \frac{3k^{*}\lambda_{2}}{sb}+\left(1+\frac{3s}{\lambda_{2}}\right)\epsilon . \end{array}$$

This completes the proof of the Theorem.
